# Care needs and care consumption in psychosis: a 4-year longitudinal analysis of guideline concordant care

**DOI:** 10.1017/S2045796021000640

**Published:** 2021-11-19

**Authors:** L. Roebroek, J. Bruins, D. Roe, P. Delespaul, S. de Jong, A. Boonstra, E. Visser, S. Castelein

**Affiliations:** 1 Lentis Psychiatric Institute, Lentis Research, Groningen, The Netherlands; 2 University Medical Center Groningen, University Center for Psychiatry, Rob Giel Research Center, University of Groningen, Groningen, The Netherlands; 3 Faculty of Behavioural and Social Sciences, University of Groningen, Groningen, The Netherlands; 4 Department of Community Mental Health, University of Haifa, Haifa, Israel; 5 Faculty of Psychiatry & Psychology, Maastricht University, Maastricht, The Netherlands; 6 Mondriaan Mental Health Trust, Heerlen-Maastricht, The Netherlands; 7 Faculty of Economics and Business, University of Groningen, Groningen, The Netherlands

**Keywords:** Evidence-based care, psychiatry, psychotic disorders, routine outcome monitoring

## Abstract

**Aims:**

People with psychotic disorders receive mental healthcare services mainly for their psychiatric care needs. However, patients often experience multiple physical or social wellbeing-related care needs as well. This study aims to identify care needs, investigate their changes over time and examine their association with mental healthcare consumption and evidence-based pharmacotherapy.

**Methods:**

This study combined annually obtained routine outcome monitoring (ROM) data with care consumption data of people with a long-term psychotic illness receiving treatment in four Dutch mental healthcare institutes between 2012 and 2016. Existing treatment algorithms were used to determine psychiatric, physical and social wellbeing-related care needs based on self-report questionnaires, semi-structured interviews and physical parameters. Care consumption was measured in hours of outpatient mental healthcare consumption per year. Generalised estimating equation models were used to calculate odds ratios of care needs and their associations with time, mental healthcare consumption and medication use.

**Results:**

Participants (*n* = 2054) had on average 7.4 care needs per measurement and received 25.4 h of care per year. Physical care needs are most prevalent and persistent and people with more care needs receive more mental healthcare. Care needs for psychotic symptoms and most social wellbeing-related care needs decreased, whereas the chance of being overweight significantly increased with subsequent years of care. Several positive associations were found between care needs and mental healthcare consumption as well as positive relations between care needs and evidence-based pharmacotherapy.

**Conclusions:**

This longitudinal study present a novel approach in identifying care needs and their association with mental healthcare consumption and pharmacotherapy. Identification of care needs in this way based on ROM can assist daily clinical practice. A recovery-oriented view and a well-coordinated collaboration between clinicians and general practitioners together with shared decisions about which care needs to treat, can improve treatment delivery. Special attention is required for improving physical health in psychosis care which, despite appropriate pharmacotherapy and increasing care consumption, remains troublesome.

## Introduction

### Care needs

Psychotic disorders are characterised by symptoms such as hallucinations, delusions, disorganised thinking, poverty of speech, apathy and social withdrawal, which may be severe and persistent (Borelli and Solari, 2019). Finding effective treatment for psychosis-related symptoms can be challenging (Torres-Gonzalez *et al*., 2014). Up to one-third of people with a psychotic illness experience persistent negative symptoms (Kirschner *et al*., 2017). Nearly half are faced with comorbid depression and substance abuse at some point during their life, with obsessive compulsive disorders and anxiety being present in 12 and 15% of the people, respectively (Buckley *et al*., 2009; Achim *et al*., 2011). With regards to physical health, cardio-metabolic risk factors are highly prevalent with half of the people with a psychotic illness meeting the criteria for metabolic syndrome (Bruins *et al*., 2017). These physical risk factors contribute to a two- to three-fold excess mortality rate compared to the general population (De Hert *et al*., 2011). With regards to social wellbeing, loneliness is very common, potentially worsening psychotic symptoms (Michalska da Rocha *et al*., 2017). Homelessness and a lack of daytime activities are additional issues affecting social wellbeing (Thornicroft *et al*., 2004; Mitchell *et al*., 2011). In an attempt to highlight these existing psychiatric symptoms, physical risk factors and issues affecting social wellbeing during clinical encounters, a treatment algorithm was developed which conceptualises these factors into different care needs (Tasma *et al*., 2018). Care needs which remain unmet, meaning that patients do not receive any form of treatment for these needs, are strong predictors of reduced quality of life for people with severe mental illness (Mojtabai *et al*., 2009; Nevarez-Flores *et al*., 2019). Several of these unmet needs tend to persist over subsequent years (Mäkinen *et al*., 2008; Buckley *et al*., 2009; Achim *et al*., 2011; Mitchell *et al*., 2011), emphasising both the difficulty and importance of providing adequate treatment. The aforementioned conceptualisation of care needs enables investigation of the prevalence of these needs in a large psychiatric population and their relation with provided care.

### Evidence-based care consumption

In 2012, the second Dutch multidisciplinary guideline for schizophrenia was released (Alphen *et al*., 2012), followed in 2018 by the standard of care for psychosis (Care standard psychosis, 2018), both largely in line with National Institute for Health and Care Excellence (NICE) guidelines from the UK (National Institute for Health and Care Excellence, 2014). These guidelines contain advice, recommendations and instructions for assessment, diagnosis and treatment of people with psychotic disorders (Alphen *et al*., 2012; National Institute for Health and Care Excellence, 2014; Care standard psychosis, 2018). Guideline concordant psychosis care can reduce symptoms, hospitalisation and mortality rates in these patients (Miller *et al*., 2004; Janssen *et al*., 2005; Cullen *et al*., 2013). Studies suggest that despite their apparent utility, adherence to clinical guidelines in regular mental healthcare is suboptimal (Bauer, 2002; Girlanda *et al*., 2017). For example, 60% of people with a psychotic illness diagnosed with metabolic disorders did not receive any form of guideline-recommended treatments for their condition (Bruins *et al*., 2017). Furthermore, two-thirds of the care needs of patients with psychotic disorders in Dutch mental healthcare were not reflected in their treatment plans (Tasma *et al*., 2016, 2017). This could, in part, be explained by insufficient resources, such as a lack of recommended interventions and trained practitioners in regional care (van Weeghel *et al*., 2011). Another explanation could be that clinicians sometimes struggle to correctly assess all of their patients’ needs. Routine outcome monitoring (ROM) is a method of using standard instruments to systematically monitor patients’ health and wellbeing over time (Trauer, 2010). It can be helpful in identifying care needs and providing input for a collaborative decision-making process. ROM also has the potential to monitor changes in these needs over time. It is important to get a better understanding of the relation between targeted evidence-based mental healthcare consumption and care needs of patients with a psychotic illness in order to offer optimal treatment. In this study, we will combine longitudinal ROM data with care consumption data and use existing treatment algorithms to identify care needs. Next, we will investigate how interventions and treatments offered in daily clinical practice are related to these care needs and how they develop over subsequent years.

### Research aim

The first aim of this study is to systematically describe the prevalence of psychiatric, physical and social wellbeing-related care needs of people with psychotic disorders. The second aim is to study changes in their care needs over subsequent years. The final aim is to explore the relationship between targeted evidence-based mental healthcare consumption and pharmacotherapy with care need outcomes.

## Method

### Data and participants

Data were obtained from an ongoing ROM cohort, called the Pharmacotherapy Monitoring and Outcome Survey (PHAMOUS), which screens people receiving care in various mental healthcare institutions in Northern Netherlands on a yearly basis (Bartels-Velthuis *et al*., 2018). All patients with a psychotic disorder (DSM-5 diagnoses: 295.90, 295.40, 295.70, 297.1, 298.8 or 298.9) were selected. Data included were limited to the yearly screenings between 2012 and 2016, because data on the care patients received from 2017 and onwards were not yet available at the time of analysis due to a new registration approach. People with a minimum of two consecutive screenings within a 9-to-15-month interval were included. For the analyses of longitudinal changes, participants had a minimum of two and a maximum of five assessments. Four institutions agreed to participate. The Medical Ethical Committee of the University Medical Center Groningen (UMCG) confirmed that anonymised PHAMOUS data can be used for scientific research (Research registration number 201700763, 9 January 2018). The procedures of this study were in accordance with local legislation and the Declaration of Helsinki.

### Care need measures and algorithms

Three domains of care were assessed: psychiatric symptoms, physical health and social wellbeing. Each domain contained subcategories adding to a total of 23 care needs (see online Supplementary Appendix 1).

### Psychiatric care needs

Eight psychiatric symptoms were derived from the Positive and Negative Syndrome Scale (PANSS) (Kay *et al*., 1987), a semi-structured interview assessing clinical remission and the clinician-rated Health of the Nation Outcome Scale (HoNOS) (Pirkis *et al*., 2005), containing 12 items ranging from 0 (no problem) to 4 (severe problem).

### Physical care needs

A total of eight physical care needs were defined. We used the Subject Response to Antipsychotics questionnaire (SRA-34) (Wolters *et al*., 2006), a self-report questionnaire measuring (side) effects of pharmacotherapy with 34 items on a 3-point scale (1 = no, 2 = yes, to some extent and 3 = yes, to a large extent). Physical parameters (i.e. blood pressure, body mass index and waist circumference) and a blood sample (glucose, haemoglobin A1c, low-density lipoprotein cholesterol, triglycerides and prolactin) were used to assess physical care needs (Bartels-Velthuis *et al*., 2018).

### Social wellbeing care needs

A total of seven care needs regarding social wellbeing were extracted from the HoNOS and Manchester Short Assessment of Quality of Life (ManSA; Priebe *et al*., 1999), a self-report questionnaire with 16 items on a 7-point Likert scale ranging from 1 (could not be worse) to 7 (could not be better).

An overview of the 23 care needs is listed in Table 2. (Combinations of) cut-off scores for all aforementioned instruments were used to calculate care needs as binary indicators (see online Supplementary Appendix 1 for a more detailed overview). These cut-off scores were based on existing validated algorithms from guidelines and/or consensus from expert panel discussion groups which included psychiatrists, psychologists, nurse-practitioners and researchers (Tasma *et al*., 2018).

### Care consumption measures and evidence-based pharmacotherapy

Care consumption data were derived from the registration of diagnosis-related groups (DRGs). DRGs include all invoiced mental healthcare consumption from individual patients. For this study, DRG data were combined with PHAMOUS data by an external third party to guarantee an anonymised merged data file. First, the duration (in h) of outpatient mental healthcare consumption per year was computed for every patient. In order to specify a proportion of evidence-based care consumption (in h), an expert panel of 20 clinicians filled out an online questionnaire to determine which type of DRG care qualifies as evidence-based care for each care need (see online Supplementary Appendix 2). Evidence-based pharmacotherapy was also dichotomised (see online Supplementary Appendix 3) into present or absent for every applicable care need based on recommendations from the Dutch multidisciplinary guideline for schizophrenia (Alphen *et al*., 2012), care standard for psychosis (Care standard psychosis, 2018) and guidelines for specific care needs [e.g. the guideline for cardiovascular risk management Drenthe or the Dutch multidisciplinary guideline for depression (Spijker *et al*., 2013)].

### Analysis

Descriptive analyses were used to compare sociodemographic and clinical characteristics of the study sample with the overall PHAMOUS population (Bartels-Velthuis *et al*., 2018). A multilevel analysis was conducted to estimate a model predicting care consumption based on the total amount of care needs. The associations between individual care needs and care consumption were analysed with generalised estimating equation (GEE) models (Liang and Zeger, 1986). This method extends the generalised linear model for clustered data and allows for correlations between repeated measures of individuals over time when analysing within and between-subjects’ relationships (Heck *et al*., 2012). The models were constructed using binomial logistic regression with an exchangeable correlation structure and a robust estimation of variance (Heck *et al*., 2012). Every care need acted as a dichotomous dependent variable in separate logistic GEE analyses. Moment of assessment (i.e. 1, 2, 3, 4 or 5) was added as a scale weight variable in the GEE analyses to map the natural development of care needs over time (Heck *et al*., 2012). Care consumption (i.e. the total number of provided hours of mental healthcare per year) was also added as a scale variable and evidence-based pharmacotherapy was added as a dichotomous factor with medication being prescribed as the reference category (no evidence-based (EVB)-medication = 0 and EVB-medication = 1). Odds ratios were calculated for every care need and the predictors (i.e. time, care consumption and medication) were added to the GEE analyses to provide an indication of their effects on the change in odd ratio from one measurement to the next. Data from the SRA and several physical parameters were used to calculate care needs for anticholinergic side effects, sexual dysfunction and smoking. These data were not imputed because they were not missing at random. Subsequently, a smaller sample size was used when calculating care needs for anticholinergic side effects (*n* = 2395), sexual dysfunction (*n* = 2330) and smoking (*n* = 1335). Multiple imputations with predictive mean matching were used for imputing the other missing scale data for the HoNOS, PANSS, ManSA and the physical parameters of PHAMOUS (see Table 1). A total of 15 imputed datasets were generated and combined using Rubin's rule (Rubin, 2004). The impact of the imputation on the results was evaluated by comparing the pooled effects to the effects of the original dataset. All statistical analyses were carried out against the 0.01 significance level and performed using the Statistical Package of the Social Sciences (SPSS), version 27 (IBM, 2021).

**Table 1.** Demographics of participants (*n* = 2054)

Demographics	Mean (s.d.) or % (*n*)
Age (years)	51.0 (11.3)
Gender: male	62.4 (1152)
Illness duration (years)	17.2 (14.4)
Diagnosis	% (*n*)
Schizophrenia	55.2 (1133)
Schizoaffective disorder	12.8 (264)
Substance induced	12.2 (251)
Psychosis not otherwise specified (NOS)	2.5 (52)
Delusional disorder	2.0 (40)
Schizophreniform disorder	1.6 (33)
Definitive diagnosis missing	13.7 (281)
Number of care needs	Mean (s.d.)
Psychiatric (s.d.) (range 0–8)	1.7 (1.3)
Physical (s.d.) (range 0–8)	3.9 (1.4)
Social-wellbeing (s.d.) (range 0–7)	1.7 (1.6)
Total (s.d.) (range 0–23)	7.4 (2.8)
Care consumption	Mean (s.d.)
Yearly care consumption in hours	25.4 (27.2)

s.d., standard deviation.

## Results

### Sample characteristics

**Fig. 1.**
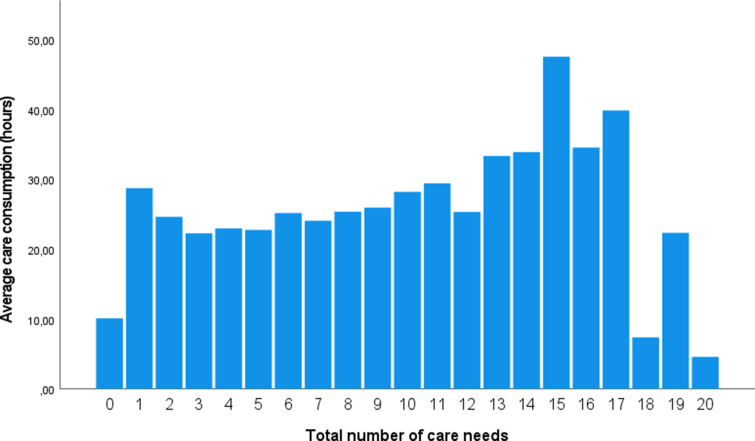
Total care needs and average care consumption.

A total of 2054 participants met the inclusion criteria for this study, who participated in a total of 5277 assessments (60.6% had two assessments, 22.1% had three assessments, 17.0% had four assessments and 0.3% had five assessments). The demographic characteristics of this sample are presented in Table 1 and are comparable to the overall PHAMOUS population (Bartels-Velthuis *et al*., 2018), except for the slightly higher average age in this study (51 *v*. 45 years). Participants had, on average, 7.4 out of the 23 care needs per measurement. Care need percentages ranged from 1.9% for self-harm to 90.2% for bodyweight (see Table 2). Physical needs accounted for more than half of all care needs, with bodyweight (90.2%), hyperlipidaemia (81.8%) and smoking (62.9%) being the most prevalent ones. Participants received, on average, 25.4 h of mental health care per year (s.d. 27.2). The number of care needs positively predicted the amount of care consumption (*F*_(1, 5277)_ = 523 997, *p* < 0.001) (see Fig. 1). Participants’ predicted care consumption is equal to 18.8 + 0.86 × (*x* care needs) hours of care consumption.

**Table 2.** Percentage of patients with care needs (dichotomised) in all measurements (*n* = 5277)

	% (*n*)
Psychiatric care needs	
Positive symptoms	54.3
Negative symptoms	50.1
Substance use	23.8
Depressive symptoms	23.4
Anxiety	11.9
Agitation	6.6
Compulsive symptoms	2.6
Self-harm	1.9
Physical care needs	
Bodyweight	90.2
Hyperlipidaemia	81.8
Smoking^a^	62.9
Anticholinergic side effects^b^	62.1
Hypertension	58.2
(Pre)diabetes type II	54.5
Sexual dysfunction^c^	44.7
Movement disorder	42.3
Social-wellbeing care needs	
Social relationships	48.9
Sexuality	29.5
Housing conditions	22.5
Daytime activities	21.9
Intimacy	21.2
Personal safety	14.7
Family support	13.3

a Smoking (*n* = 1335).

b Anticholinergic side effects (*n* = 2395).

c Sexual dysfunction (*n* = 2330).

### Association between care needs and time

The likelihood of experiencing psychiatric care needs remained the same on every measurement (*M* = 53.3 weeks) for most needs (see Table 3). However, the likelihood of experiencing positive [*β* = −0.080, 95% confidence interval (CI) (−0.138 to −0.021)] or negative symptoms [*β* = −0.077, 95% CI (−0.134 to −0.021)] decreased significantly with every measurement. The likelihood of being overweight [*β* = 0.240, 95% CI (0.130 to 0.351)] significantly increased with every measurement, whereas the likelihood of experiencing other physical care needs did not. The likelihood of experiencing social wellbeing-related care needs changed the most, with the likelihood of having a care need for social relationships [*β* = −0.079, 95% CI (−0.138 to −0.020)], housing conditions [*β* = −0.108, 95% CI (−0.189 to −0.027)], daytime activities [*β* = −0.102, 95% CI (−0.176 to −0.029)] and personal safety [*β* = −0.150, 95% CI (−0.247 to −0.053)] significantly decreasing with every measurement.

**Table 3.** Odds ratios of having a care need and associations with time (*M* = 53.3 weeks, s.d. 5.7), mental healthcare consumption and evidence-based pharmacotherapy in psychotic disorders

	*β* (OR)	*β* Time	*p*	*β* Consumption	*p*	*β* Medication	*p*
Psychiatric care needs							
Positive symptoms	0.151 (1.16)	−0.080	0.008*	0.003	0.033	0.176	0.009*
Negative symptoms	0.148 (1.16)	−0.077	0.008*	−0.002	0.180	0.061	0.383
Substance use	−1.125 (0.33)	−0.031	0.373	0.001	0.544	0.024	0.780
Depressive symptoms	−1.323 (0.27)	−0.092	0.018	0.007	0.000**	0.170	0.073
Anxiety	−2.398 (0.09)	−0.073	0.165	0.006	0.001*	0.693	0.000**
Agitation	−2.608 (0.07)	−0.118	0.079	0.006	0.003*	0.077	0.591
Compulsive symptoms	−3.586 (0.03)	0.057	0.512	−0.005	0.251	0.537	0.009*
Self-harm	−3.805 (0.02)	−0.286	0.044	0.012	0.000**	−0.075	0.808
Physical care needs							
Bodyweight	1.508 (4.52)	0.240	0.000**	0.008	0.001*	0.355	0.006*
Hyperlipidaemia	1.837 (6.28)	−0.055	0.183	−0.002	0.129	−0.295	0.003*
Hypertension	0.329 (1.39)	0.011	0.703	−0.002	0.116	0.175	0.059
(Pre)diabetes type II	−0.039 (0.96)	0.086	0.014	−0.003	0.047	1.468	0.000*
Anticholinergic side effects^a^	0.567 (1.76)	−0.076	0.081	0.004	0.035	0.021	0.798
Sexual dysfunction^b^	−0.255 (0.78)	−0.022	0.597	0.003	0.050	−0.021	0.816
Smoking^c^	−0.306 (0.74)	−0.006	0.870	0.000	0.899		
Movement disorder	0.509 (1.66)	0.035	0.500	−0.002	0.363		
Social-wellbeing care needs							
Social relationships	0.019 (1.02)	−0.079	0.008*	0.004	0.002*		
Sexuality	−0.876 (0.42)	−0.045	0.189	0.004	0.005*		
Intimacy	−1.361 (0.26)	−0.024	0.507	0.004	0.006*		
Housing conditions	−1.105 (0.35)	−0.108	0.009*	0.003	0.022		
Daytime activities	−1.236 (0.29)	−0.102	0.006*	0.005	0.000**		
Personal safety	−1.585 (0.21)	−0.150	0.003*	0.004	0.004*		
Family support	−1.986 (0.14)	−0.017	0.706	0.006	0.000**		

OR, odds ratio.

*p < 0.01, **p < 0.001.

a Anticholinergic side effects (*n* = 2395).

b Sexual dysfunction (*n* = 2330).

c Smoking (*n* = 1335).

### Association between care needs and care consumption

Mental healthcare consumption was positively associated with half of all the psychiatric care needs (see Table 3): the likelihood of experiencing depressive symptoms [*β* = 0.007, 95% CI (0.004–0.009)], anxiety [*β* = 0.006, 95% CI (0.003–0.009)], agitation [*β* = 0.006, 95% CI (0.002–0.010)] and self-harm [*β* = 0.012, 95% CI (0.007–0.017)] increased with more hours of mental healthcare consumption. For physical care needs only the likelihood for being overweight [*β* = 0.008, 95% CI (0.003–0.013)] increased significantly with more hours of mental healthcare consumption. The likelihood of experiencing social wellbeing care needs changed most with social relationships [*β* = 0.004, 95% CI (0.001–0.007)], sexuality [*β* = 0.004, 95% CI (0.001–0.007)], intimacy [*β* = 0.004, 95% CI (0.001–0.007)], daytime activities [*β* = 0.005, 95% CI (0.002–0.008)], personal safety [*β* = 0.004, 95% CI (0.001–0.007)] and family support [*β* = 0.006, 95% CI (0.002–0.008)], increasing significantly with more hours of mental healthcare consumption.

### Association between care needs and evidence-based care and pharmacotherapy

When examining the association between evidence-based pharmacotherapy (see online Supplementary Appendix 3) and psychiatric care needs, the likelihood of experiencing anxiety [*β* = 0.680, 95% CI (0.492–0.895)] and compulsive symptoms [*β* = 0.586, 95% CI (0.138–1.1034)] was significantly increased in people who received some form of medication for those specific care needs (see Table 3). For physical care needs the likelihood of being overweight [*β* = 0.355, 95% CI (0.099–0.610)] and having (pre)diabetes type II [*β* = 1.468, 95% CI (1.235–1.671)] increased in people that received some form of medication for those specific care needs. Conversely, the likelihood of having hyperlipidaemia [*β* = −0.295, 95% CI (−0.490 to −0.100)] significantly decreased in people using medication for hyperlipidaemia (see Table 3). Differentiation of care consumption into evidence-based and other care consumption for every specific care need yielded no significant results (see online Supplementary Appendix 2).

The original data and pooled data were compared in order to test the impact on the outcomes (online Supplementary S2). Deltas between the pooled effects and the effects of the original dataset across full models varied between *β* = 0.002 and *β* = 0.075 indicating an adequate imputation.

## Discussion

This study distinguished 23 different care needs in three domains: psychiatric needs (eight needs), physical needs (eight needs) and social wellbeing needs (seven needs). Participants had, on average, 7.4 care needs per measurement of which more than half were physical. This is in line with previous research showing increased cardio-metabolic risks in people with a psychotic illness (De Hert *et al*., 2011), which in part can be attributed to long-term use of antipsychotic medication (Bak *et al*., 2014). The prevalence of physical care needs in this study was relatively high compared to previous studies, examples being high bodyweight (90.2 *v.* 49.4%), hypertension (58.2 *v.* 38.7%) and smoking (62.9 *v.* 54.3%) (Mitchell *et al*., 2011). The prevalence rates did not change over subsequent years, except for an increasing chance of being overweight, thereby suggesting the nature of most physical care needs is persistent. The absence of significant relationships between physical care needs and mental healthcare consumption might be due to the majority of care consumption being psychiatric and psychosocial interventions (see online Supplementary Appendix 2). Ideally, clinicians collaborate with general practitioners to address these physical needs which can also take place outside of mental healthcare settings such as community centres, gyms or assisted living accommodations. However, this is often not the case for people in psychosis care (Tasma *et al*., 2016, 2017). No clear relation was found between evidence-based pharmacotherapy for specific physical care needs and a decrease of these needs over subsequent years, which suggests that treatment with pharmacotherapy alone might not be enough to address these needs.

Our findings about psychiatric care needs are more positive compared to physical care needs, as participants averaged 1.8 needs per measurement, with positive (54.33%) and negative (50.1%) symptoms as the most common needs. The chance of experiencing these core symptoms of a psychotic illness significantly decreased with every subsequent year. This is an interesting finding, since negative symptoms tend to be persistent and difficult to treat (Mäkinen *et al*., 2008). Comorbidity with other psychiatric symptoms such as anxiety, substance abuse, depressive and compulsive symptoms was present in less than a quarter of participants, which is comparable to previous findings (Mäkinen *et al*., 2008; Buckley *et al*., 2009; Achim *et al*., 2011). These needs are more persistent over time as chances for these psychiatric needs did not significantly decrease over time. Interestingly, pharmacotherapy was positively associated with an increased chance for some psychiatric care needs. It is important to note that our medication algorithm does not account for polypharmacy and overmedication which could be a potential explanation for these observed associations. When focusing on social wellbeing, participants averaged 1.7 care needs per measurement, with social relationships (48.9%) and sexuality (29.5%) as the most mentioned needs. Loneliness and a lack of meaningful relationships and intimacy are key issues affecting social wellbeing for people with a psychotic illness (Michalska da Rocha *et al*., 2017). The chances for most needs surrounding social wellbeing decreased over subsequent years, potentially indicating a more transient and less persistent nature compared to physical or psychiatric care needs. For example, needs surrounding personal safety or housing conditions might be prioritised during treatment because they are more acute. It is also possible people get accustomed to being alone over time.

In the Netherlands, people with less severe mental health issues generally receive care in basic mental health services, whereas people with a severe mental illness tend to receive care in specialised mental healthcare services (Kroneman *et al*., 2016). Participants in this study received, on, average 25 h of outpatient specialised mental healthcare per year, which is about double the amount of care for people in basic mental healthcare services (Kroneman *et al*., 2016). Contrary to previous findings (Drukker *et al*., 2007), more care needs were associated with more mental healthcare consumption. This could, in part, explain the positive associations between a higher chance of several psychiatric and social wellbeing care needs and more care consumption. In other words, participants received more care when they had more identified care needs, which is reflected by the increased chances of having these specific care needs on subsequent measurements.

### Clinical implications

Appropriately allocating care in mental health care services among a diverse population is considered by some as the most important academic challenge of modern day mental healthcare (Wykes *et al*., 2015). This study attempts to contribute to this challenge by proposing a methodology for identifying care needs and studying their relation with care consumption. Our results confirm earlier findings in which people with psychotic disorders are often faced with multiple persistent physical care needs, accompanied by one or both core symptoms of psychosis and a need for social connection and intimacy. These findings justify investing in lifestyle or social wellbeing-related interventions such as peer support groups in the form of eating clubs (Vogel *et al*., 2019). At an individual level, care needs identified by ROM can serve as useful input during consultations. When they are combined with treatment recommendations, for example by using a computerised clinical decision aid, they have the potential to facilitate shared-decision making and help patients and mental health care workers to decide together on a course of treatment (Roebroek *et al*., 2020). Our analyses can also be utilised to assess needs and care provisions in teams or institutions. For example, in this study a majority of care needs are of a physical nature, yet only a small fraction of the provided care is specifically aimed to treat those conditions (see online Supplementary Appendix 2). It is important to note that a perfect fit between identified care needs and appropriate care, in which all needs are addressed in treatment, is likely not feasible, considering the amount of comorbidity as demonstrated in this study. Moreover, identified care needs might not always be perceived as an actual need by patients, which makes the implementation of treatment interventions a strategic choice, ideally collaboratively explored and decided on by clinicians and patients together.

### Strengths and limitations

A strength of this study is the innovative way in which ROM was used to identify care needs and monitor their changes over time, potentially acting as an example for other institutions and future research. With over 2000 participants yielding more than 5000 measurements over a 4-year period, this study features a unique clinical sample. There has been a tendency in previous research to focus on either psychiatric symptoms or cardiometabolic risk factors. To the best of our knowledge, this is the first study in psychosis research attempting to identify a broad range of potential care needs and their longitudinal development on the psychiatric, physical as well as social wellbeing-related domain. By combining ROM data with care consumption data, this study was able to identify various associations between care needs and (evidence-based) care consumption. An important limitation of this study is that our care consumption data only include outpatient mental healthcare consumption. The degree to which they apply also to inpatient settings is yet to be studied. Moreover, because people with more care needs tend to consume more care these associations have to be interpreted with caution. This study was not set up to investigate prospective associations, so it remains unclear if for instance more care needs lead to more care consumption or vice versa. Some care needs such as bodyweight and blood pressure are known to be correlated with age. Due to the limitations of a cohort study, it could not be determined how much of this increase was beyond what can be expected in the general population over time. This study is focused on patients receiving long-term psychiatric care, given the diagnostic criteria and the inclusion of only people with multiple measurements. This is also reflected in the higher mean age of participants included in this study compared to the overall PHAMOUS population (51 *v*. 45 years), which should be taken into account when generalising these results, for instance when comparing them to first-episode populations. It is also important to note that we used a clinical conceptualisation of care needs, identified with existing treatment algorithms and based on ROM data. Only part of the data was obtained by self-report questionnaires and therefore does not always take into account the subjective experience of needs. For example, some participants might smoke or be overweight without perceiving this as an issue needing treatment. The identification and conceptualisation of care needs serves a clinical purpose, but a collaborative effort based on shared decision-making is needed. Future research could opt to conceptualise care needs differently, for example on continuous scales, potentially making the analyses more sensitive to change (Wiersma *et al*., 2009).

## Conclusion

This longitudinal study identified psychiatric, physical and social wellbeing-related care needs with existing treatment algorithms based on yearly obtained ROM data combined with care consumption data. Physical care needs were most prevalent and persistent. Positive and negative symptoms were the most common psychiatric care needs, but the chance of experiencing these needs decreased with subsequent years of care. Care needs related to social wellbeing had a more transient character. As might be expected, people with the highest needs received the most mental healthcare potentially explaining the positive relation between several of these needs with care consumption. The prime focus in psychosis care used to be on recovery of psychiatric symptoms but is shifting more towards recovery-oriented care encompassing both personal recovery and social wellbeing (Anthony, 1993). Ideally, the responsibility for physical care should be an interplay between clinicians and general practitioners. Defining and identifying care needs based on ROM has the potential to assist daily clinical practice and help institutions with care allocation in order to accommodate people's care needs on these different domains.

## Data Availability

The datasets used in this study will be made available when requested to the corresponding author.

## Supplementary material

For supplementary material accompanying this paper visit http://dx.doi.org/10.1017/S2045796021000640. click here to view supplementary material
