# Supplementary material for: Care needs and care consumption in psychosis: a 4-year longitudinal analysis of guideline concordant care
Source: Epidemiol Psychiatr Sci. 2021 Nov 19;30:e73. doi: 10.1017/S2045796021000640 (PMC8611928; doi:10.1017/S2045796021000640)
Supplement: Supplementary file 1 [file epssup.zip › S2045796021000640sup001.docx]

| **Description** | **Care Needs** |
| --- | --- |
| Electroconvulsive therapy | Positive symptoms, depressive symptoms |
| Creative therapy | Negative symptoms, depressive symptoms, agitation, anxiety, self-harm, social relationships |
| Activating counseling | Positive symptoms, negative symptoms, depressive symptoms, substance abuse, anxiety, social relationships |
| Physiotherapy | Bodyweight, (pre)diabetes type II, movement disorder |
| Behavioral therapy | Positive symptoms, depressive symptoms, anxiety, OCD |
| Light therapy | Depressive symptoms |
| Psychotherapy | Depressive symptoms, anxiety, OCD, self-harm |
| Communicative treatment | Negative symptoms, depressive symptoms, anxiety, social relationships, intimacy, family support |
| Psychomotor therapy | Negative symptoms, depressive symptoms, anxiety, social relationships, self-harm, personal safety, positive symptoms |
| System Therapy | Depressive symptoms, social relationships, intimacy, family support |
| Cognitive behavioral therapy | Positive symptoms, negative symptoms, depressive symptoms, anxiety, OCD |

Appendix 2. Diagnosis related group codes for applicable care needs
