# Supplementary material for: Care needs and care consumption in psychosis: a 4-year longitudinal analysis of guideline concordant care
Source: Epidemiol Psychiatr Sci. 2021 Nov 19;30:e73. doi: 10.1017/S2045796021000640 (PMC8611928; doi:10.1017/S2045796021000640)
Supplement: Supplementary file 1 [file epssup.zip › S2045796021000640sup002.docx]

| **Care Needs** | **Medication** | **Evidence-based medication steps** |
| --- | --- | --- |
| Positive symptoms | aripiprazole, bromopride, chlorprothixene, clozapine, flupentixol, haloperidol, levropromazine, olanzapine, periciazine, perphenazine, pipamperone, pimozide, promazine hydrochloride, quetiapine, risperidone, sulpiride, triflupromazine, zuclopenthixol, bromopride, fluphenazine, flupentixol, fluspirileen, penfluridol | 1) increase dose antipsychotic 2) switch to different antipsychotic  3) start clozapine 4) addition antipsychotic to clozapine  5) addition lamotrigine, memantine or lithium to clozapine |
|  | aripiprazole, bromopride, chlorprothixene, clozapine, flupentixol, haloperidol, levropromazine, olanzapine,  periciazine, perphenazine, pipamperone, pimozide, promazine hydrochloride, quetiapine, risperidone, sulpiride, triflupromazine, zuclopenthixol, bromopride, fluphenazine, flupentixol, fluspirileen, penfluridol | 1) reduce dose antipsychotic 2) polypharmacy reduction  3) switch to antipsychotic with lower D2 affinity 4) addition antidepressant 5) addition lamotrigine to clozapine |
| Substance use | aripiprazole, clozapine, olanzapine, paliperidone, quetiapine, risperidone | 1) second generation antipsychotic |
| Depressive symptoms | imipramine, amitriptyline, clomipramine, nortriptyline, venlafaxine, mirtazapine, citalopram, fluoxetine,  fluvoxamine, paroxetine, sertraline, trazodone, moclobemide, tranylcypromine, phenelzine, escitalopram,  duloxetine, bupropion, hyperiplant | 1) antidepressant 2) Reduce dose antipsychotic  3) switch to antipsychotic with lower D2 affinity |
| Anxiety | lorazepam, bromazepam, chlordiazepoxide, midazolam, clobazam, oxazepam, prazepam,  temazepam, lormetazepam, nitrazepam, zolpidem, lorazepam, bromazepam, chlordiazepoxide, midazolam | 1) anxiety medication |
| Agitation | lorazepam, clozapine | 1) lorazepam and or clozapine |
| Compulsive symptoms | clomipramine, fluoxetine, citalopram, escitalopram, fluvoxamine, paroxetine, sertraline, venlafaxine | 1) compulsive symptoms medication |
| Self-harm | clozapine | 1) clozapine |
| Bodyweight | aripiprazole, amisulpride, ziprasidone, haloperidol, pimozide, perphenazine, metformin | 1) switch to high potent first generation antipsychotic 2) metformin |
| Hyperlipidemia | lipid modifying agents | 1) lipid modifying agents 2) no olanzapine, clozapine or quetiapine |
| Hypertension | antihypertension medication | 1) antihypertension medication |
| (Pre)diabetes type II | antihyperglycemics, metformin | 1) antihyperglycemics 2) metformin |
| Anticholinergic side  effects | aripiprazole, bromopride, chlorprothixene, clozapine, flupentixol, haloperidol, levropromazine, olanzapine,  periciazine, perphenazine, pipamperone, pimozide, promazine hydrochloride, quetiapine, risperidone, sulpiride, triflupromazine, zuclopenthixol, bromopride, fluphenazine, flupentixol, fluprofen, penfluridol | 1) reduce dose antipsychotic 2) switch to different antipsychotic |
| Sexual dysfunction | aripiprazole, bromopride, chlorprothixene, clozapine, flupentixol, haloperidol, levropromazine, olanzapine,  periciazine, perphenazine, pipamperone, pimozide, promazine hydrochloride, quetiapine, risperidone, sulpiride, triflupromazine, zuclopenthixol, bromopride, fluphenazine, flupentixol, fluprofen, penfluridol | 1) reduce dose antipsychotic  2) switch to antipsychotic with lower D2 affinity |

Appendix 3. Medication algorithm
