# Supplementary material for: Care needs and care consumption in psychosis: a 4-year longitudinal analysis of guideline concordant care
Source: Epidemiol Psychiatr Sci. 2021 Nov 19;30:e73. doi: 10.1017/S2045796021000640 (PMC8611928; doi:10.1017/S2045796021000640)
Supplement: Supplementary file 1 [file epssup.zip › S2045796021000640sup003.docx]

| **Psychiatric care needs** | **Instruments** | **Cut-off Scores** |
| --- | --- | --- |
| Positive symptoms | PANSS (P1,P2, P3, P4, P5, P6 & P7), HoNOS | P1>3, P2>2, P3>3, P4>4, P5>4, P6>3, P7>3. P1,P3,P4,P5,P6<P7: min 4 items score 3. HoNOS>1. P1-P7 if 1 or more>3. P1,P2,P3,P6 minimum 2 items score 3. P1-P7 min 3 items 3. HoNOS>2. In case≥1 or more of these apply |
| Negative symptoms | PANSS (N1, N2, N3, N4, N5, N6 & N7) | N1>3, N4>3, N6>3. Both N1,N4,N6=3. N1-N7 if 1 or more>3. If 2 items of N1,N2,N3,N4 score 3. N1-N7 min 3 items  score 3. In case ≥1 or more of these apply |
| Substance Use | HoNOS (item 3) | A6>3. HoNOS>1. In case≥1 or more of these apply |
| Depressive symptoms | PANSS (A6), HoNOS (item 7) | HoNOS> 2 & C |
| Anxiety | PANSS (A2), HoNOS (item 8) | HoNOS> 0 & < 3. HoNOS > 2 |
| Agitation | HoNOS (item 1) | HoNOS> 1 |
| Compulsive symptoms | HoNOS (item 8) | A2 > 3, HoNOS > 2 & B. In case ≥ 1 or more of these apply |
| Self-harm | HoNOS (item 2) | HoNOS > 1 |
| **Physical care needs** | **Instruments** | **Cut-off Scores** |
| Bodyweight | SRA (item 3 & 32), BMI, abdominal circumference | SRA, item 3: score 1 or 2, item 32 score 1 or 2. BMI > 25 kg/m². AC > 88 cm (f), > 102 cm (m) |
| Hyperlipidemia | Low-density lipoprotein (lab test) | LDL ≥ 2,5 mnol/L and/or TG N > 2,2 mnol/L |
| Smoking | Anamnesis | Yes |
| Anticholinergic side effects | SRA (item 9, 12, 16 & 20) | SRA, if ≥ 1 score of 2 or if ≥ 2 score of 1 |
| Hypertension | Blood pressure (lab test) | Blood pressure ≥ 130/85 |
| (Pre)diabetes type II | Glucose (lab test) | Glucose ≥ 5,6 and/or hemoglobin A1c > 39 (>5,7%) |
| Sexual dysfunction | SRA (item 15, 21, 35) | SRA, item 15: score 1 or 2, item 21: score 1 or 2, item 35: score 1 or 2 (f). In case ≥ 1 or more of these apply |
| Movement disorder | Movement test, SRA (item 18 & 28) | Movement check diagnosis: dyskinesia, dystonia, parkinsonism, akathisia. SRA, item 18: score 1 or 2, item 28: score 1 or 2 |
| **Social-wellbeing care needs** | **Instruments** | **Cut-off Scores** |
| Social relationships | ManSA (item 12), HoNOS (item 9) | ManSA < 4, HoNOS > 1. In case ≥ 1 or more of these apply |
| Sexuality | ManSA (item 15) | ManSA < 4 |
| Housing conditions | ManSA (item 2 & 3), HoNOS (item 11) | ManSA, item 2 < 4, item 3 < 4, HoNOS > 1. In case ≥ 1 or more of these apply |
| Daytime activities | ManSA (item 4), HoNOS (item 12) | ManSA < 4, HoNOS > 1. In case ≥ 1 or more of these apply |
| Intimacy | ManSA (item 14) | ManSA < 4 |
| Personal safety | ManSA (item 7, 8 & 9) | ManSA, item 7 = yes, item 8 < 4, item 9 = yes |
| Family support | ManSA (item 13) | ManSA < 4 |

PANSS = Positive and negative symptom scale (semi-structured interview), HoNOS = Health of the nation outcome scale (clinician-rated), SRA = Subject response to antipsychotic questionnaire (self-report), ManSA = Manchester short assessment of quality of life (self-report)
